# Loneliness and emotional support helpline use in Spain: a 20-year observational study

**DOI:** 10.3389/fpsyg.2026.1852702

**Published:** 2026-07-13

**Authors:** Rogelio Hernández−Díaz, Alejandra Aguilar-Latorre, Carmen Vicente-García, Eva Pilar Chueca-Miguel, Rosa Magallón-Botaya

**Affiliations:** 1 Department of Medicine, Psychiatry and Dermatology, Faculty of Medicine, University of Zaragoza, Zaragoza, Spain; 2 Torrero Primary Care Health Center - Zaragoza II Sector, Aragonese Health Service, Zaragoza, Spain; 3 Institute for Health Research Aragón (IIS Aragón), Zaragoza, Spain; 4 Department of Psychology and Sociology, Faculty of Human Sciences and Education, University of Zaragoza, Huesca, Spain; 5 Research Network on Chronicity, Primary Care and Health Promotion RD24/0005/0004, RICAPPS, Madrid, Spain; 6 Department of Psychology and Sociology, Faculty of Social Sciences and Humanities, University of Zaragoza, Teruel, Spain; 7 Department of Physiatry and Nursing, Faculty of Health Sciences, University of Zaragoza, Zaragoza, Spain; 8 Parkinson's Association of Aragon, Zaragoza, Spain

**Keywords:** emotional support helpline, help-seeking, loneliness, psychological distress, registry-based study

## Abstract

**Background:**

Emotional support helplines provide low-threshold assistance to people experiencing psychological distress, yet long-term evidence on reasons for contact remains limited in Spain. This study aimed to describe the distribution of psychological motives for contacting Teléfono de la Esperanza between 2004 and 2023, with particular attention to loneliness, and to examine temporal, sex-, and age-related patterns.

**Methods:**

We conducted a retrospective observational study using anonymized routine registry data from the International Association Teléfono de la Esperanza (ASITES). The dataset included 2,194,175 contact records registered between 2004 and 2023. Descriptive analyses summarized annual volume, caller characteristics, and primary presenting problems. Annual proportions were calculated to examine temporal patterns. Associations between caller characteristics and selected primary presenting motive were examined using multinomial logistic regression, with loneliness/communication difficulties as the reference outcome category and sex, age group, and calendar year entered simultaneously as predictors. Adjusted odds ratios, 95% confidence intervals, and model-based adjusted predicted probabilities were reported.

**Results:**

Contact volume increased over time, reaching its highest levels in 2021–2023. Most contacts were by telephone, and most callers were women (67.1%); age distribution was concentrated in midlife. Loneliness/communication difficulties was the most frequent primary presenting problem among the selected motives examined (10.1%), followed by depressed mood (7.2%) and anxiety-related problems (7.0%). In the multinomial model, age group showed the largest contribution to model fit. Adjusted predicted probabilities showed a strong age gradient for loneliness/communication difficulties, increasing from 15.75% among contacts aged ≤18 years to 77.44% among those aged ≥76 years. In contrast, suicidality-related motives were concentrated in younger groups. Men had higher adjusted relative odds of suicidal ideation and suicidal crisis than women, whereas women had higher relative representation of depressed mood and grief/bereavement.

**Conclusion:**

Helpline contact records reflect distinct age-, sex-, and time-related patterns of distress. Loneliness emerged as a major reason for contact, particularly in later life, supporting the value of helplines as an accessible public mental health resource and as a potential gateway for targeted prevention and referral strategies.

## 1 Introduction

Mental health problems represent a major public health challenge in contemporary societies and account for a substantial share of disability and unmet care needs worldwide. At the same time, many forms of emotional distress are shaped not only by individual vulnerability but also by broader social environments, including loneliness, social disconnection, and difficulties in accessing timely support. In this context, formal mental health systems do not always fully cover the demand for immediate, accessible, and low-threshold emotional support, and community-based and volunteer-led initiatives may play an important complementary role (Patel et al., 2023; World Health Organization, 2021, 2022, 2025).

Emotional support and crisis helplines are an important component of public mental health systems, offering immediate, low-threshold support to people experiencing psychological distress. These services commonly provide brief, single-session interventions oriented toward crisis de-escalation, emotional regulation, and—when needed—suicide prevention, while also addressing a broader range of acute suffering related to anxiety, depressed mood, relational conflict, traumatic experiences, and other psychosocial stressors (Mazzer et al., 2021; Mental Health, Brain Health and Substance Use (MSD), WHO, 2018). In many settings, helplines function as front-line responses that can reduce immediate distress and facilitate linkage to formal and informal sources of care (Vaghela et al., 2025).

A defining feature of many helplines is that they can be accessed confidentially and, in some cases, anonymously. Anonymity may reduce perceived stigma and social-evaluative threat, facilitating disclosure of sensitive experiences and potentially enabling earlier or more candid help-seeking.

Evidence from mental health screening research suggests that anonymous formats can increase reporting of mental health symptoms and suicidal ideation in some populations (Warner, 2011), although findings vary depending on the population and the outcomes assessed (Fear et al., 2012). For helplines, confidentiality and ease of access are therefore central to their public health value—particularly for individuals who are geographically isolated, socially disconnected, or reluctant to engage with formal services due to stigma or practical barriers (Mental Health, Brain Health and Substance Use (MSD), WHO, 2018).

The service landscape has also evolved: while helplines were historically telephone-based, many now integrate email, chat, and other digital modalities, which may better align with user preferences and widen reach (Vaghela et al., 2025). Alongside these shifts, outcome-focused research continues to support the perceived usefulness of crisis calls among suicidal callers; for example, recent evaluations of crisis line outcomes show high proportions of callers reporting that the call was helpful and contributed to improved safety during the crisis episode (Gould et al., 2025).

Empirical evidence from telephone counseling and crisis helpline services also suggests that helplines are sensitive to broader societal disruptions. During the COVID-19 pandemic, studies conducted within the Austrian nationwide telephone crisis counseling service 142 (TelefonSeelsorge) showed that loneliness and mental health concerns were among the most frequently reported topics by callers, and that these issues were perceived by counselors as having increased compared with the pre-pandemic period (Humer et al., 2021). A subsequent longitudinal study of TelefonSeelsorge counselors similarly identified loneliness and mental health as the most common caller problems at two pandemic-period assessment points, reinforcing the relevance of helpline data for monitoring psychosocial needs during prolonged periods of social disruption (Humer et al., 2022).

Emotional support and crisis helplines are now widely established across countries and health systems, including large-scale national services and long-standing community-based organizations. This broad international presence underlines their relevance as accessible first-contact resources for people experiencing distress. Within Spain, one of the best-known and longest-standing examples is *Teléfono de la Esperanza*.

*Teléfono de la Esperanza* is a long-standing, volunteer-based emotional support service providing listening, guidance, and psychosocial accompaniment. Its model emphasizes active listening and supportive engagement, offering an accessible entry point for individuals who may not otherwise seek formal mental health care (Ruiz, 2014). Beyond its service role, the systematic recording of call-related information creates an opportunity to study population needs and patterns of distress using real-world data generated in routine practice. At the organizational level, activity reports illustrate sustained high demand (e.g., annual call volumes publicly reported by the organization) and underscore the potential value of analyzing service data to inform prevention and service planning.

One psychological concern that is especially relevant for helpline use is loneliness. Loneliness is commonly defined as an aversive state that occurs when there is a perceived discrepancy between the social relationships a person desires and those they believe they currently have (Heinrich and Gullone, 2006; World Health Organization, 2025). Importantly, loneliness has moved to the forefront of public health agendas: the WHO Commission on Social Connection highlights loneliness as widespread globally and calls for urgent action to strengthen social connection as a determinant of health (World Health Organization, 2025).

A large body of research links loneliness and related forms of social disconnection to poorer mental and physical health, quality of life, and longevity. Meta-analytic evidence indicates that loneliness and social isolation are associated with increased mortality risk (Holt-Lunstad et al., 2015), while WHO syntheses emphasize impacts across the life course and across regions (World Health Organization, 2025). Beyond these long-term associations, loneliness is also closely intertwined with common mental health problems—particularly depression and anxiety—and may act as an amplifier of distress under conditions of social disruption (Heinrich and Gullone, 2006; Yanguas et al., 2018).

Research attention to loneliness intensified during and after the COVID-19 pandemic, with European longitudinal and panel evidence documenting marked fluctuations in loneliness early in the pandemic and heterogeneity across time and population groups (Köster and Lipps, 2024; Rebechi et al., 2024). In Spain, recent studies also document substantial loneliness among older adults and identify demographic and contextual factors associated with unwanted loneliness (Dolz-del-Castellar et al., 2025; Hernández-López et al., 2024). Against this backdrop, emotional support services may function as a critical outlet for individuals seeking immediate interpersonal connection, validation, and coping support when informal networks are disrupted or insufficient.

Loneliness may also be relevant to patterns of repeated help-seeking and frequent service use. Research on people who frequently contact emergency medical services has found high levels of social loneliness, poverty, and low quality of life, suggesting that recurrent contact with urgent services may partly reflect unmet psychosocial and social-care needs rather than acute clinical problems alone (Agarwal et al., 2019). Qualitative evidence from frequent ambulance service users further indicates that loneliness and social isolation are complex, persistent experiences that may contribute to repeated contacts with emergency services when other sources of support are unavailable, insufficient, or difficult to access (Moseley et al., 2024). Although emotional support helplines differ from emergency medical services, this literature is relevant because it highlights how loneliness may contribute to repeated demand for low-threshold, immediate-contact services.

The Spanish context is particularly relevant because unwanted loneliness has been shown to carry a substantial social and economic burden. Recent estimates suggest that the tangible costs associated with unwanted loneliness in Spain reached approximately 14.1 billion euros in 2021, equivalent to around 1.2% of national GDP, including both healthcare costs related to increased service use and medication consumption, and productivity losses due to reduced working time and premature mortality (Casal et al., 2025). These findings support the need to examine loneliness not only as an individual psychological experience, but also as a public health issue with implications for health-service planning, prevention, and community-based support.

Despite a growing international literature on crisis services, published evidence based on large-scale, long-term helpline registries remains limited in Spain, particularly regarding loneliness as a presenting motive. To our knowledge, few studies have examined how the reasons for contacting these services evolve over time, how patterns of distress may shift across historical periods, or whether these motives differ systematically by sex and age. Call-log studies from other contexts show that reasons for contacting helplines are diverse and include suicidality, mood and affective difficulties, and family or relationship problems (Turkington et al., 2020). Moreover, emerging work using helpline chat/call data suggests that loneliness is frequently interwoven with depression and suicidality and can shift in salience during major societal stressors (Grimland et al., 2026). This gap is especially relevant for loneliness, given its growing public health importance and its close links with depression, anxiety, and suicidality.

Understanding these presenting motives—and how they differ across groups and over time—can inform service planning and targeted prevention strategies. Gender differences are particularly relevant, as epidemiological patterns in internalizing symptoms and suicidal behavior often vary by gender, and help-seeking pathways may differ across the lifespan (Kiely et al., 2019). In addition, age gradients in loneliness are a recurrent concern in population research and have been highlighted as a policy priority, including among adolescents and older adults (Hosozawa et al., 2025; World Health Organization, 2025). Examining whether loneliness and other psychological motives differ by gender and age in helpline contacts can therefore help align service responses with the needs of diverse users.

### 1.1 Objectives

The primary objective of this study was to describe the distribution of psychological motives for contacting *Teléfono de la Esperanza* using a large anonymized registry of contact records from 2004 to 2023, with particular attention to loneliness as a presenting motive. Secondary objectives were: (1) to describe temporal patterns in presenting motives across the study period, and (2) to examine whether reasons for contacting the service differed by sex and age group, with special attention to contacts in which loneliness was recorded as the main presenting motive.

## 2 Materials and methods

### 2.1 Study design and data source

We conducted a retrospective observational study using anonymized routine registry data from the International Association Teléfono de la Esperanza (ASITES), a long-standing emotional support and crisis helpline service in Spain. The dataset included all available contacts recorded between 2004 and 2023. The data were generated during routine service activity monitoring and were not originally collected for research purposes. Each record corresponds to one contact episode registered by the service. Data access and use were established through a collaboration agreement between ASITES and the Aragón Primary Care Research Group (GAIAP), defining confidentiality obligations, purpose limitation, restricted access, non-commercial use, and the prohibition of re-identification attempts.

### 2.2 Unit of analysis and study population

The unit of analysis was the individual contact record. Therefore, the study describes contact episodes rather than unique individuals. Because the data were anonymized and contained no personal identifiers, repeated contacts by the same person could not be linked across the registry. Consequently, it was not possible to determine whether an individual contacted the service more than once, including multiple times within the same day. All available contact records registered between 2004 and 2023 were eligible for inclusion in the descriptive characterization of the registry. Records were excluded from specific analyses only when they lacked valid data for the variables required for that analysis.

### 2.3 Data extraction, eligibility criteria, and preprocessing

The original registry dataset was reviewed to identify the variables required for the present study, including year of contact, sex, age group, contact modality, and primary presenting problem. Records were checked for valid year values within the study period and for valid coding of the main variables used in the analyses. Contacts with missing year were excluded from annual analyses, and contacts with missing primary presenting problem were excluded from analyses involving presenting motives.

The primary presenting problem field included both psychological presenting motives and other contact-related categories. Some categories explicitly denoted non-informative or non-analytical contacts, such as prank calls, incomplete calls, silent calls, or out-of-hours contacts. These categories were not interpreted as psychological presenting motives and were not included among the selected outcomes of interest. However, unless otherwise specified, annual descriptive denominators were based on all contacts with a valid primary presenting problem in order to describe the distribution of selected motives within the full set of valid registered contacts.

Secondary and tertiary presenting-problem fields were available only for a subset of contacts and showed substantial missingness. Therefore, inferential analyses focused on the primary presenting problem, which was the most consistently completed field and the most appropriate variable for describing the main reason for contact.

### 2.4 Measures

The registry included: (1) basic caller characteristics, particularly sex and age group; (2) temporal information, including year and month of contact; (3) contact characteristics such as service location/center and contact modality (e.g., telephone, in-person at the center, email, or online chat); (4) additional contextual information available for a subset of records, including marital status, living arrangement, caller frequency, and geographic origin; and (5) a structured classification of the main presenting problem recorded for each contact, with the possibility of recording secondary and tertiary problems in a minority of cases. Consistent with the aims of this paper, analyses focused on caller sex, caller age group, calendar year, and the primary presenting problem recorded by the service.

### 2.5 Outcomes

The primary outcome for the regression-based analyses was selected primary presenting motive, modeled as a seven-category multinomial outcome. The categories were loneliness/communication difficulties, depressed mood/depression, anxiety-related problems, grief/bereavement, suicidal ideation, suicidal crisis, and suicide act in progress. Loneliness/communication difficulties was used as the reference outcome category in the multinomial models.

For descriptive analyses, loneliness/communication difficulties was also examined as a primary motive of particular interest, given the aims of the study. The remaining selected motives were used to contextualize loneliness within other relevant reasons for contacting the helpline.

These outcomes were not assessed using standardized diagnostic instruments or validated questionnaires. Rather, they were based on the primary presenting problem recorded in routine practice by helpline counselors according to the service’s documentation categories. Therefore, the outcomes should be interpreted as counsellor-recorded presenting motives for contact, not as clinical diagnoses or validated symptom measures. Potential variability in counsellor documentation and interpretation is considered in the limitations of the study.

### 2.6 Missing data

Some variables showed missingness because they were not systematically completed for all contacts, particularly additional sociodemographic or contextual fields. For the main analyses, we prioritized variables with high completeness, namely sex, age group, year of contact, and primary presenting problem. Missing values were handled using an available-case approach. Records were included in each analysis when they had valid data for the variables required for that specific analysis.

Thus, analyses involving age group excluded contacts with missing age group, analyses involving sex excluded contacts with missing sex, and annual analyses excluded contacts with missing year or missing primary presenting problem. No imputation was performed because the study was based on routinely collected registry data. The corresponding denominators are reported for each analysis.

### 2.7 Statistical analysis

We first described contact volume across calendar years and summarized caller and contact characteristics using frequencies and percentages. Percentages were calculated using valid denominators for each variable.

To address temporal dynamics across the 20-year observation period, we calculated annual proportions of selected primary presenting problems from 2004 to 2023. For each calendar year, the denominator was the number of contacts with a valid primary presenting problem. Annual percentages were calculated for loneliness/communication difficulties, depressed mood, anxiety-related problems, grief/bereavement, suicidal ideation, suicidal crisis, and suicide act in progress. These analyses were descriptive and were used to examine temporal patterns without assuming that the full observation period was homogeneous.

We then examined associations between caller characteristics and selected primary presenting motive using multinomial logistic regression. The outcome included seven mutually exclusive categories: loneliness/communication difficulties, depressed mood, anxiety-related problems, grief/bereavement, suicidal ideation, suicidal crisis, and suicide act in progress. Loneliness/communication difficulties was used as the reference outcome category. Sex, age group, and calendar year were entered simultaneously as predictors. Calendar year was included as a categorical covariate to adjust for temporal variation across the observation period. Exact age was not available in the anonymized registry; therefore, age was modeled using the predefined age-group variable.

The main multinomial model included all contacts whose primary presenting problem corresponded to one of the seven selected motives and who had complete data on sex, age group, and calendar year. Adjusted odds ratios (aORs) and 95% confidence intervals were reported. Model fit was assessed using likelihood-ratio tests comparing the final model with the intercept-only model, together with Akaike information criterion, Bayesian information criterion, −2 log likelihood, and pseudo-R^2^ indices.

A sex-by-age-group interaction was tested directly. Because the full seven-category interaction model produced unstable estimates and warnings related to the Hessian matrix, we evaluated the interaction in a sensitivity multinomial model excluding the rarest outcome category*, suicide act in progress*. This sensitivity model included six outcome categories and the same predictors as the main model, with the addition of the sex-by-age-group interaction term.

To aid interpretation, we also derived model-based adjusted predicted probabilities from the main seven-category multinomial model. These probabilities were averaged within sex, age group, and sex-by-age-group strata and expressed as percentages. They were used to describe absolute differences in predicted motive distributions across caller groups.

Missing values were handled using an available-case approach. Records were included in each analysis when they had valid data for the variables required for that specific analysis. Thus, analyses involving age group excluded contacts with missing age group, analyses involving sex excluded contacts with missing sex, and annual analyses excluded contacts with missing year or missing primary presenting problem. No imputation was performed because the study was based on routinely collected registry data. The corresponding denominators are reported for each analysis.

Given the very large sample size, statistical significance was not interpreted in isolation. Interpretation emphasized effect sizes, confidence intervals, predicted probabilities, and temporal patterns rather than *p*-values alone. For the regression-based analyses, we prespecified aORs ≥1.25 or ≤0.80 as the smallest effect size of interest. For adjusted predicted probabilities, absolute differences of at least 2 percentage points were considered potentially meaningful. All tests were two-tailed with *α* = 0.05. Analyses were conducted using IBM SPSS Statistics, version 29.0 (IBM Corp., Armonk, NY, USA).

### 2.8 Ethical and data protection considerations

The study was conducted using anonymized registry data provided by ASITES. The research protocol was reviewed and approved by the Clinical Research Ethics Committee of Aragón (CEIC Aragón, CEICA), which issued a favorable opinion on 22 February 2023 (reference PI23/078; dated 10/02/2023). Data processing complied with the General Data Protection Regulation (EU) 2016/679 and Spanish Organic Law 3/2018. Access to the dataset was restricted to authorized research personnel, and the collaboration agreement prohibited re-identification attempts and unauthorized data sharing.

## 3 Results

### 3.1 Registry size, caller demographics, and contact characteristics (2004–2023)

The registry comprised 2,194,175 contact records between 2004 and 2023, with year information available for 2,194,150 records (25 missing). Regarding caller demographics, sex was recorded for 2,123,738 contacts (96.8% of all records). Among those with sex information, 67.1% were women (1,424,050) and 32.9% were men (699,688). Age group was recorded for 1,973,141 contacts (89.9%); the distribution was concentrated in midlife, particularly among callers aged 36–45 years (25.6%), 46–55 years (25.0%), and 56–65 years (18.9%).

**Figure 1:**
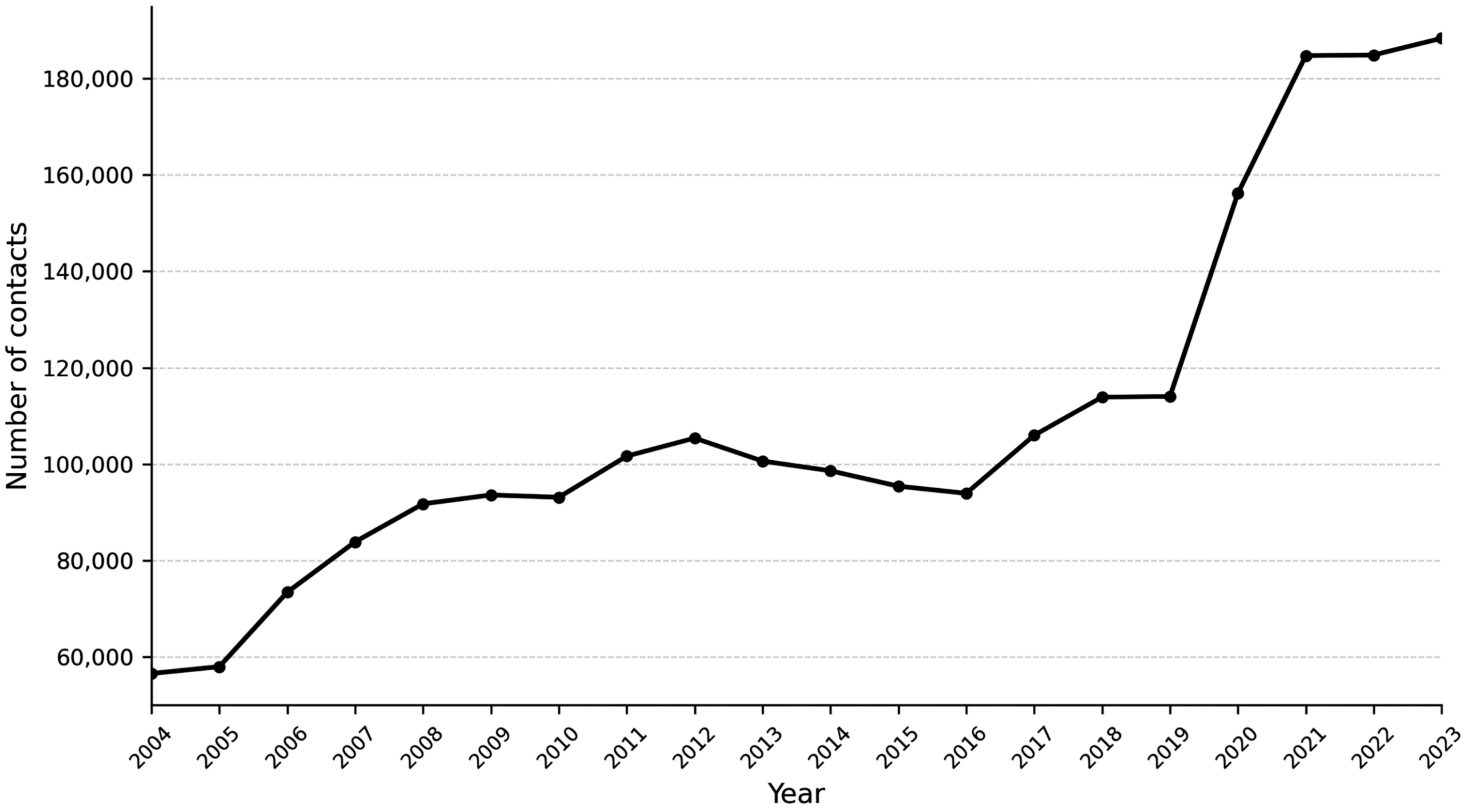
Annual number of helpline contacts recorded by *Teléfono de la Esperanza*, 2004–2023.

Annual contact volume increased over time, with the highest activity observed in the most recent years (e.g., 156,168 contacts in 2020 [7.1%], 184,739 in 2021 [8.4%], 184,866 in 2022 [8.4%], and 188,330 in 2023 [8.6%]) (Figure 1). Most contacts were conducted by telephone (2,110,645; 96.2%), whereas 39,347 (1.8%) were recorded as in-person at the center. Smaller proportions involved email (14,836; 0.7%) and other modalities, including online chat and specific programs (Table 1).

**Table 1:** Descriptive characteristics of helpline contacts (ASITES), 2004–2023.

Domain	Category	*n*	%
Registry size (Year available)	Total records with year (valid)	2,194,150	100.0
	Missing year	25	—
	Total records (overall)	2,194,175	—
Annual volume (% of total with year)	2004	56,582	2.6
	2005	58,002	2.6
	2006	73,440	3.3
	2007	83,890	3.8
	2008	91,766	4.2
	2009	93,606	4.3
	2010	93,127	4.2
	2011	101,666	4.6
	2012	105,401	4.8
	2013	100,639	4.6
	2014	98,619	4.5
	2015	95,427	4.3
	2016	93,945	4.3
	2017	105,981	4.8
	2018	113,915	5.2
	2019	114,041	5.2
	2020	156,168	7.1
	2021	184,739	8.4
	2022	184,866	8.4
	2023	188,330	8.6
Contact modality	Total with modality (valid)	2,194,072	100.0
	Telephone	2,110,645	96.2
	In-person at center	39,347	1.8
	Email	14,836	0.7
	Other modalities/programs (combined)*	29,244	1.3
	Missing modality	103	—
Caller sex	Total with sex (valid)	2,123,738	100.0
	Women	1,424,050	67.1
	Men	699,688	32.9
	Missing sex	70,437	—
Caller age group	Total with age group (valid)	1,973,141	100.0
	≤18 years	25,927	1.3
	19–25 years	80,482	4.1
	26–35 years	296,573	15.0
	36–45 years	504,149	25.6
	46–55 years	493,076	25.0
	56–65 years	373,056	18.9
	66–75 years	140,901	7.1
	≥76 years	58,977	3.0
	Missing age group (system missing)	221,034	—
Primary presenting problem (selected; % of valid primary problem field)	Total with primary problem (valid)	2,192,728	100.0
	Loneliness/communication difficulties	221,686	10.1
	Depressed mood	158,534	7.2
	Anxiety-related problems	153,544	7.0
	Grief/bereavement	19,595	0.9
	Suicidal ideation	30,128	1.4
	Suicidal crisis	8,600	0.4
	Suicide act in progress	2,110	0.1
	Missing primary problem	1,447	—

*Other modalities/programs include (sum): online intervention, helpline chats, and program-specific contact channels recorded in the registry.

Percentages are calculated using valid denominators within each domain (e.g., sex, age group, contact modality, and primary presenting problem). Missing values are reported as absolute numbers only and are not included in percentage calculations.

### 3.2 Distribution of primary presenting problems

The primary presenting problem was recorded for nearly all contacts (minimal missing). The most frequently recorded primary reasons included loneliness and communication difficulties (221,686; 10.1%), depressed mood (158,534; 7.2%), and anxiety-related problems (153,544; 7.0%). Suicidality-related presentations were less frequent as primary reasons: suicidal ideation (30,128; 1.4%), suicidal crisis (8,600; 0.4%), and a suicide act in progress (2,110; 0.1%). Grief/bereavement was recorded in 19,595 contacts (0.9%).

Given the high missingness in secondary and tertiary problem fields, inferential analyses focused on the primary presenting problem.

### 3.3 Annual trends in selected presenting motives, 2004–2023

**Figure 2:**
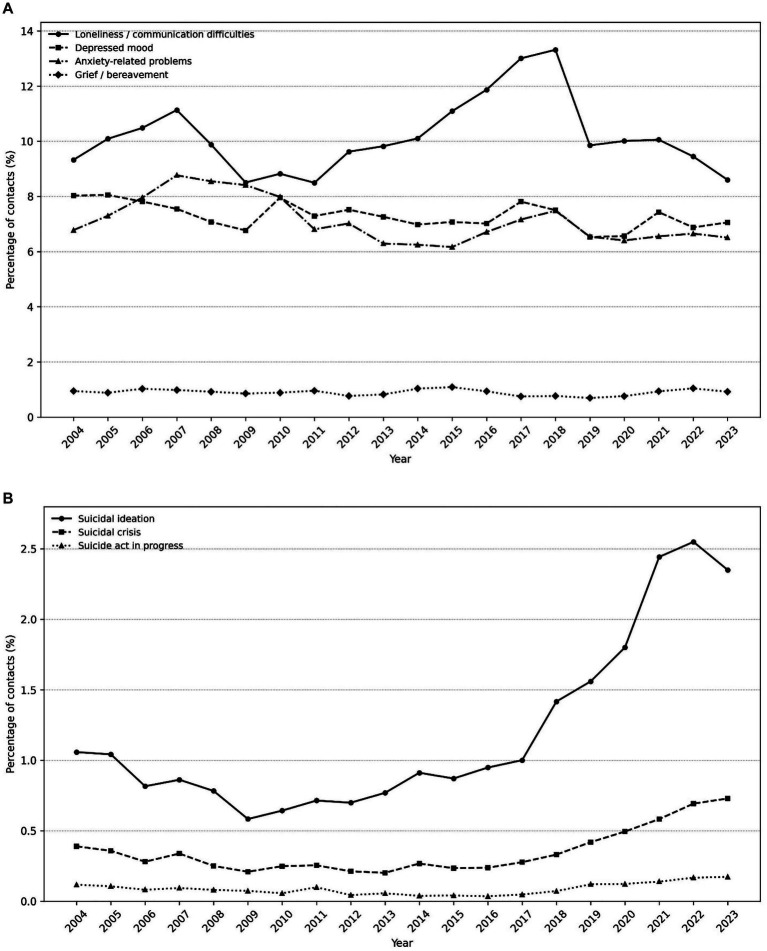
Annual distribution of selected presenting motives, 2004–2023. Annual distribution of selected primary presenting motives among contacts with a valid primary presenting problem, 2004–2023. Panel **(A)** shows loneliness/communication difficulties, depressed mood, anxiety-related problems, and grief/bereavement. Panel **(B)** shows suicidality-related motives: suicidal ideation, suicidal crisis, and suicide act in progress. Percentages were calculated using the annual number of contacts with a valid primary presenting problem as the denominator.

To address temporal dynamics across the 20-year observation period, we examined the annual distribution of selected primary presenting motives among contacts with a valid primary presenting problem. The annual percentage of loneliness/communication difficulties fluctuated across the study period, ranging from 8.5% in 2011 to 13.3% in 2018 (Figure 2A; Supplementary Table S1). Depressed mood and anxiety-related problems showed comparatively smaller annual variation, with depressed mood ranging from 6.5 to 8.1% and anxiety-related problems from 6.2 to 8.8%. Grief/bereavement remained consistently infrequent, ranging from 0.7 to 1.1% of annual contacts.

Suicidality-related motives represented a smaller proportion of annual contacts but showed a clearer increase in the most recent years (Figure 2B; Supplementary Table S1). Suicidal ideation ranged from 0.6% in 2009 to 2.5% in 2022, while suicidal crisis increased from a minimum of 0.2% in 2013 to 0.7% in 2023. Suicide act in progress remained rare across the whole period, although its annual percentage was highest in 2023 (0.17%).

### 3.4 Multinomial logistic regression of selected primary presenting motive

A multinomial logistic regression model was fitted among contacts whose primary presenting problem corresponded to one of the seven selected motives and who had complete data on sex, age group, and calendar year (*N* = 575,811). Loneliness / communication difficulties was used as the reference outcome category. The model including sex, age group, and calendar year improved fit compared with the intercept-only model, χ^2^ (162) = 65,706.67, *p* < 0.001, AIC = 18,157.43, BIC = 20,049.71, −2 log likelihood = 17,821.43, with Nagelkerke R^2^ = 0.115. Likelihood-ratio tests indicated that age group made the largest contribution to model fit, χ^2^ (42) = 48,540.55, *p* < 0.001, followed by calendar year, χ^2^ (114) = 12,394.51, *p* < 0.001, and sex, χ^2^ (6) = 3,272.14, *p* < 0.001. Adjusted odds ratios and 95% confidence intervals for the main multinomial model are shown in Table 2.

**Table 2:** Multinomial logistic regression predicting selected primary presenting motive.

Predictor	Depressed mood	Anxiety-related problems	Grief/bereavement	Suicidal ideation	Suicidal crisis	Suicide act in progress
Sex						
Men vs. women	0.804 (0.792–0.816)	0.870 (0.857–0.883)	0.624 (0.602–0.647)	1.468 (1.430–1.506)	1.391 (1.329–1.455)	1.165 (1.063–1.276)
Age group						
≤18 vs. ≥ 76 years	11.946 (10.961–13.019)	15.725 (14.320–17.267)	5.958 (5.024–7.066)	95.100 (80.322–112.597)	106.710 (76.347–149.149)	823.110 (203.707–3325.902)
19–25 vs. ≥ 76 years	11.030 (10.395–11.703)	17.655 (16.508–18.882)	4.714 (4.168–5.331)	56.951 (48.684–66.621)	74.491 (53.989–102.776)	441.816 (109.928–1775.718)
26–35 vs. ≥ 76 years	8.303 (7.897–8.730)	15.207 (14.331–16.136)	3.547 (3.199–3.933)	26.030 (22.331–30.343)	31.123 (22.626–42.809)	161.040 (40.124–646.339)
36–45 vs. ≥ 76 years	6.513 (6.204–6.838)	10.439 (9.849–11.064)	3.062 (2.773–3.381)	15.968 (13.711–18.597)	21.737 (15.826–29.856)	101.977 (25.431–408.918)
46–55 vs. ≥ 76 years	5.220 (4.974–5.478)	7.430 (7.011–7.874)	2.972 (2.695–3.278)	10.960 (9.411–12.764)	14.360 (10.452–19.728)	59.092 (14.727–237.112)
56–65 vs. ≥ 76 years	3.636 (3.463–3.817)	4.680 (4.414–4.962)	2.552 (2.312–2.816)	5.912 (5.068–6.895)	6.635 (4.813–9.147)	23.496 (5.827–94.745)
66–75 vs. ≥ 76 years	2.280 (2.165–2.402)	2.686 (2.524–2.858)	1.824 (1.641–2.027)	2.372 (2.011–2.797)	2.651 (1.883–3.732)	6.086 (1.441–25.699)

Values are adjusted odds ratios (aORs) with 95% confidence intervals. *N* = 575,811. The reference outcome category was loneliness/communication difficulties. Sex was coded as men vs women, with women as the reference category. Age group was entered categorically, with ≥76 years as the reference category. The model was adjusted for calendar year, with 2023 as the reference year. Calendar-year coefficients are not shown for parsimony. The prespecified smallest effect size of interest was aOR ≥1.25 or ≤0.80. Given the large sample size, interpretation should focus on effect sizes and confidence intervals rather than *p* values alone.

Using loneliness / communication difficulties as the reference category, men had lower adjusted relative odds than women of grief/bereavement (aOR = 0.624, 95% CI [0.602, 0.647]) and higher adjusted relative odds of suicidal ideation (aOR = 1.468, 95% CI [1.430, 1.506]) and suicidal crisis (aOR = 1.391, 95% CI [1.329, 1.455]), with these effects exceeding the prespecified smallest effect size of interest. The sex difference for depressed mood was close to the prespecified threshold but did not exceed it based on the point estimate, while differences for anxiety-related problems and suicide act in progress were smaller in magnitude.

Age gradients were pronounced. Compared with contacts aged ≥76 years, younger age groups had substantially higher adjusted relative odds of depressed mood, anxiety-related problems, grief/bereavement, and suicidality-related motives rather than loneliness / communication difficulties. These age-related differences were particularly large for suicidal ideation, suicidal crisis, and suicide act in progress. For example, compared with contacts aged ≥76 years, those aged ≤18 years had substantially higher relative odds of suicidal ideation (aOR = 95.100, 95% CI [80.322, 112.597]), suicidal crisis (aOR = 106.710, 95% CI [76.347, 149.149]), and suicide act in progress (aOR = 823.110, 95% CI [203.707, 3325.902]) rather than loneliness / communication difficulties. These estimates should be interpreted as relative odds of each motive compared with loneliness / communication difficulties, not as absolute risks.

Observed descriptive distributions in the analytical sample are provided in Supplementary Table S2.

### 3.5 Adjusted predicted probabilities by sex and age group

Model-based adjusted predicted probabilities were derived from the main seven-category multinomial model to aid interpretation on an absolute scale. These probabilities showed a clear age gradient. The adjusted predicted probability of loneliness / communication difficulties increased steadily with age, from 15.75% among contacts aged ≤18 years to 77.44% among those aged ≥76 years. In contrast, suicidality-related motives were concentrated in younger groups. The adjusted predicted probability of suicidal ideation was 21.69% among contacts aged ≤18 years and 13.43% among those aged 19–25 years, compared with 0.97% among those aged ≥76 years.

Sex differences were more modest in absolute terms. Men had a higher adjusted predicted probability of suicidal ideation than women, whereas women had a higher adjusted predicted probability of depressed mood. The remaining sex differences were smaller in absolute magnitude. Adjusted predicted probabilities by sex and age group are reported in Supplementary Table S3.

### 3.6 Sensitivity analysis testing the sex-by-age-group interaction

A sex-by-age-group interaction was tested directly. The full seven-category interaction model produced unstable estimates and warnings related to the Hessian matrix, driven by the rarest outcome category, suicide act in progress. Therefore, the interaction was evaluated in a sensitivity multinomial model excluding this rare category.

In the six-category sensitivity model, the sex-by-age-group interaction improved model fit, χ^2^ (35) = 1,016.75, *p* < 0.001. However, the increase in Nagelkerke R^2^ was minimal, from 0.113 in the main-effects six-category model to 0.114 in the interaction model. Therefore, the seven-category main-effects model was retained as the primary model, and the interaction model was interpreted as a sensitivity analysis. Full details of the sensitivity analysis are reported in Supplementary Table S4.

## 4 Discussion

Using a large, anonymized registry of contacts to a Spanish emotional support helpline over two decades, this study describes psychological motives for help-seeking and how they vary by age, sex, and calendar year, with a particular focus on loneliness. Four main patterns stand out. First, loneliness/communication difficulties showed the clearest age gradient on the absolute scale, with adjusted predicted probabilities increasing steadily across age groups and reaching their highest level among contacts involving the oldest age group. Second, annual descriptive analyses showed that loneliness/communication difficulties fluctuated over time, while suicidality-related motives, although infrequent, showed higher proportions in the most recent years. Third, depression- and anxiety-related presenting problems were more prominent in younger and mid-adult groups, whereas suicidality-related motives were proportionally more common among adolescents and young adults. Fourth, sex differences were generally modest on the absolute scale, although men had higher adjusted relative odds of suicidal ideation and suicidal crisis than women, whereas women showed greater relative representation of depressed mood and grief/bereavement. Given the very large sample size, these findings should be interpreted primarily in terms of adjusted odds ratios, confidence intervals, predicted probabilities, and temporal patterns rather than statistical significance alone.

### 4.1 Loneliness as a key motive and its implications for service planning

The strong age gradient in loneliness aligns with broader public health and health-system perspectives that identify social disconnection as a major determinant of health in later life and a priority for health and community responses (National Academies of Sciences, Engineering, and Medicine, 2020; Office of the Surgeon General, 2023). Importantly, registry data also show that loneliness-related contacts are not confined to older adulthood: although the proportion of loneliness-related contacts rises with age, substantial numbers of loneliness contacts occur in midlife because this is where overall service use is highest. This combination (high proportion in older groups and high absolute counts in midlife) suggests two complementary needs for helplines: (1) targeted approaches for older adults (e.g., mobilizing community and health-system referral pathways, and addressing bereavement, functional limitations, or caregiving contexts that may accompany loneliness), and (2) scalable strategies for midlife callers, where loneliness may intersect with chronic stressors, relational disruption, or cumulative burden.

From an intervention perspective, evidence increasingly supports the idea that loneliness is modifiable but that “what works” may depend on mechanisms targeted. Recent systematic reviews and meta-analyses in older adults suggest that multi-component approaches (e.g., combining psychological components with skill-building or structured social connection opportunities) tend to yield more consistent benefits than single-focus approaches, although heterogeneity across studies is substantial (Duffner et al., 2024; Patil and Braun, 2024; Shekelle et al., 2024). For helplines, these findings support a practical implication: when loneliness is the presenting motive, brief crisis support may be complemented by referral options that match evidence-informed mechanisms (e.g., cognitive/behavioral approaches addressing maladaptive social cognition, structured volunteering or group programs, or coordinated social prescribing models where available). This is particularly relevant because helplines may represent one of the few low-threshold points of contact for individuals who are socially disconnected or reluctant to seek formal care.

### 4.2 Suicidality-related contacts and the role of crisis lines

Although suicidality-related motives represented a small proportion of primary presenting problems, the age pattern—higher proportions among contacts involving adolescents and young adults—fits established developmental risk gradients in suicidal thoughts and crises. The very large sample size makes even small differences statistically significant, reinforcing the need to interpret these findings in terms of proportional patterns and effect sizes. Still, even low-proportion outcomes translate into substantial absolute numbers at the population level, especially when services handle high volumes of contacts.

The annual descriptive analyses add an important temporal perspective to these findings. Although suicidality-related motives remained relatively infrequent across the registry, suicidal ideation, suicidal crisis, and suicide act in progress showed their highest annual proportions in the most recent years. These descriptive patterns should not be interpreted as causal evidence of specific external events, because the registry does not allow us to disentangle changes in population need, service visibility, recording practices, or organizational factors. However, they suggest that continued monitoring of suicidality-related contacts is warranted, particularly among younger age groups, and that helpline registries may provide useful real-world indicators of changing patterns of psychological distress over time.

These findings also matter because the broader evidence base suggests crisis lines can meaningfully reduce distress in the moment and support safety. A systematic review of crisis line effectiveness found that callers’ distress typically decreases from the beginning to the end of calls, and that specific counselor behaviors (e.g., engagement and safety-related practices) are linked to improved outcomes (Hoffberg et al., 2019). More recent evaluations of suicide prevention helpline conversations similarly show measurable improvements in crisis-related outcomes, including reductions in hopelessness and entrapment during or after contacts (e.g., helpline impact studies using pre–post designs) (Pauwels et al., 2025). Taken together, the registry patterns in this study highlight the value of ensuring robust training and quality monitoring for suicide risk assessment and management, especially for youth-related contacts and for the large midlife burden observed in absolute numbers.

### 4.3 Sex differences in motives for contacting

Women represented most contacts in the registry and showed higher representation for depressed mood and grief/bereavement, whereas men showed higher representation for suicidal ideation and suicidal crisis. These patterns broadly align with sex- and gender-linked differences observed in population mental health and help-seeking, where women more often report internalizing symptoms and engage with support services, while men show different risk profiles and help-seeking pathways (Kiely et al., 2019). At the same time, the sex effects in this registry were generally small, which is consistent with the idea that helplines serve heterogeneous users and that sex differences may reflect both underlying prevalence and differential service utilization. Practically, these results support tailoring service messaging and referral pathways to reach groups less likely to engage, and ensuring that scripts and training address gendered barriers to disclosure (e.g., stigma, norms around self-reliance) without assuming uniform needs within groups.

### 4.4 Strengths, limitations, and future directions

A key strength of this study is the scale and duration of the registry, enabling the description of presenting motives for helpline contact at a resolution that is rarely possible in survey-based or single-site studies. The 20-year observation period allowed us to examine not only age- and sex-related patterns, but also annual temporal variation in selected presenting motives. The use of routinely recorded, anonymized data also reflects real-world help-seeking behavior in a low-threshold emotional support service and provides a valuable perspective on psychosocial needs among people who actively seek support.

Several limitations should be considered. First, the unit of analysis was the contact record, not the individual person. Because the dataset was anonymized and contained no personal identifiers, repeated contacts by the same person could not be linked. As a result, frequent users may be overrepresented, and the findings should be interpreted as patterns among contact episodes rather than estimates of prevalence among unique individuals. Second, presenting problems were recorded in routine practice by helpline counselors and were not assessed using standardized diagnostic instruments or validated questionnaires. Therefore, the outcomes should be interpreted as counsellor-recorded presenting motives rather than clinical diagnoses or standardized symptom measures.

Third, routine documentation may introduce variability across counselors, centres, and calendar years. No formal inter-rater reliability assessment was available in the registry, and coding conventions, training procedures, or documentation practices may have changed during the 20-year observation period. These factors may partly influence observed differences across groups or years. Fourth, the primary presenting problem field included some categories that reflected contact characteristics rather than psychological motives, such as prank calls, incomplete calls, silent calls, or out-of-hours contacts. These categories were not interpreted as psychological presenting motives, but their presence illustrates the complexity of using routine helpline registry data for research purposes. Fifth, missingness was substantial for several contextual variables, such as marital status, living arrangement, caller frequency, and geographic origin, which constrained the analyses to the most consistently recorded fields and limited adjustment for potential confounding or subgroup profiling. Finally, because this is a service-based sample, findings describe contacts made to Teléfono de la Esperanza and should not be generalized to the prevalence of loneliness or psychological distress in the general Spanish population.

Future research could build on this work in several ways. Analytically, more advanced time-sensitive approaches, such as interrupted time-series analyses or other longitudinal modeling strategies, could examine whether major societal or organizational changes correspond to detectable shifts in presenting motives. Such analyses would require careful consideration of changes in service availability, public awareness, recording practices, and help-seeking behavior over time. Future registry systems could also consider privacy-preserving identifiers that would allow repeated contacts by the same person to be studied without compromising anonymity. Substantively, loneliness-focused work could examine the intersection of loneliness with depression and suicidality using richer co-occurrence coding where feasible, and evaluate whether referral pathways aligned with evidence-based loneliness interventions improve downstream outcomes. Finally, aligning service responses with broader health-system recommendations—such as integrating screening and referral for social isolation into care pathways—may help translate these registry findings into scalable preventive strategies.

## Data Availability

The dataset is not publicly available because it consists of anonymized registry data provided by ASITES under a collaboration agreement that restricts unauthorized sharing and prohibits re-identification attempts. Access may be considered upon reasonable request to the corresponding author and subject to prior authorization from ASITES and compliance with applicable data protection requirements. Requests to access these datasets should be directed to aaguilar@unizar.es.
