# Supplementary material for: Loneliness and emotional support helpline use in Spain: a 20-year observational study
Source: Front Psychol. 2026 Jul 13;17:1852702. doi: 10.3389/fpsyg.2026.1852702 (PMC13402487; doi:10.3389/fpsyg.2026.1852702)
Supplement: Supplementary file 1 [file Table_1.doc]

**Supplementary Table S1**

*Annual Distribution of Selected Primary Presenting Problems in Helpline Contacts, 2004-2023*

| Year | Valid contacts | Loneliness / communication difficulties | Depressed mood | Anxiety-related problems | Grief / bereavement | Suicidal ideation | Suicidal crisis | Suicide act in progress |
| --- | --- | --- | --- | --- | --- | --- | --- | --- |
| 2004 | 56,573 | 5,276 (9.33) | 4,543 (8.03) | 3,835 (6.78) | 532 (0.94) | 598 (1.06) | 220 (0.39) | 66 (0.12) |
| 2005 | 57,978 | 5,850 (10.09) | 4,670 (8.05) | 4,231 (7.30) | 511 (0.88) | 604 (1.04) | 207 (0.36) | 61 (0.11) |
| 2006 | 73,252 | 7,679 (10.48) | 5,724 (7.81) | 5,828 (7.96) | 751 (1.03) | 597 (0.81) | 205 (0.28) | 59 (0.08) |
| 2007 | 83,826 | 9,332 (11.13) | 6,328 (7.55) | 7,349 (8.77) | 822 (0.98) | 722 (0.86) | 283 (0.34) | 78 (0.09) |
| 2008 | 91,726 | 9,060 (9.88) | 6,488 (7.07) | 7,841 (8.55) | 842 (0.92) | 717 (0.78) | 229 (0.25) | 73 (0.08) |
| 2009 | 93,568 | 7,958 (8.51) | 6,333 (6.77) | 7,871 (8.41) | 796 (0.85) | 545 (0.58) | 195 (0.21) | 68 (0.07) |
| 2010 | 93,096 | 8,212 (8.82) | 7,405 (7.95) | 7,429 (7.98) | 824 (0.89) | 598 (0.64) | 231 (0.25) | 52 (0.06) |
| 2011 | 101,604 | 8,633 (8.50) | 7,406 (7.29) | 6,917 (6.81) | 968 (0.95) | 725 (0.71) | 258 (0.25) | 100 (0.10) |
| 2012 | 105,378 | 10,141 (9.62) | 7,922 (7.52) | 7,398 (7.02) | 808 (0.77) | 736 (0.70) | 223 (0.21) | 45 (0.04) |
| 2013 | 100,614 | 9,880 (9.82) | 7,305 (7.26) | 6,329 (6.29) | 823 (0.82) | 773 (0.77) | 202 (0.20) | 56 (0.06) |
| 2014 | 98,596 | 9,960 (10.10) | 6,883 (6.98) | 6,161 (6.25) | 1,015 (1.03) | 898 (0.91) | 263 (0.27) | 37 (0.04) |
| 2015 | 95,408 | 10,582 (11.09) | 6,748 (7.07) | 5,882 (6.17) | 1,035 (1.08) | 830 (0.87) | 223 (0.23) | 38 (0.04) |
| 2016 | 93,586 | 11,107 (11.87) | 6,568 (7.02) | 6,280 (6.71) | 873 (0.93) | 887 (0.95) | 222 (0.24) | 32 (0.03) |
| 2017 | 105,980 | 13,785 (13.01) | 8,278 (7.81) | 7,592 (7.16) | 789 (0.74) | 1,060 (1.00) | 293 (0.28) | 50 (0.05) |
| 2018 | 113,915 | 15,170 (13.32) | 8,546 (7.50) | 8,519 (7.48) | 870 (0.76) | 1,613 (1.42) | 376 (0.33) | 81 (0.07) |
| 2019 | 114,035 | 11,234 (9.85) | 7,442 (6.53) | 7,457 (6.54) | 788 (0.69) | 1,778 (1.56) | 477 (0.42) | 136 (0.12) |
| 2020 | 156,161 | 15,631 (10.01) | 10,250 (6.56) | 10,000 (6.40) | 1,186 (0.76) | 2,811 (1.80) | 771 (0.49) | 191 (0.12) |
| 2021 | 184,701 | 18,574 (10.06) | 13,725 (7.43) | 12,102 (6.55) | 1,719 (0.93) | 4,511 (2.44) | 1,075 (0.58) | 256 (0.14) |
| 2022 | 184,626 | 17,446 (9.45) | 12,695 (6.88) | 12,280 (6.65) | 1,918 (1.04) | 4,706 (2.55) | 1,277 (0.69) | 307 (0.17) |
| 2023 | 188,080 | 16,175 (8.60) | 13,273 (7.06) | 12,240 (6.51) | 1,725 (0.92) | 4,419 (2.35) | 1,370 (0.73) | 324 (0.17) |

*Note.* Values are n (%). Percentages are row percentages calculated using the total number of contacts with a valid primary presenting problem in each calendar year as the denominator. No primary-problem categories were excluded at this stage.
