# Supplementary material for: Loneliness and emotional support helpline use in Spain: a 20-year observational study
Source: Front Psychol. 2026 Jul 13;17:1852702. doi: 10.3389/fpsyg.2026.1852702 (PMC13402487; doi:10.3389/fpsyg.2026.1852702)
Supplement: Supplementary file 2 [file Table_2.doc]

**Supplementary Table S2**

*Observed Distribution of Selected Primary Presenting Motives by Age Group and Sex in the Analytical Sample*

Values are n (% within row).

| Group | N | Loneliness / communication difficulties | Depressed mood | Anxiety-related problems | Grief / bereavement | Suicidal ideation | Suicidal crisis | Suicide act in progress |
| --- | --- | --- | --- | --- | --- | --- | --- | --- |
| **Age group** |  |  |  |  |  |  |  |  |
| ≤18 years | 7,295 | 1,149 (15.8) | 2,049 (28.1) | 1,739 (23.8) | 227 (3.1) | 1,582 (21.7) | 396 (5.4) | 153 (2.1) |
| 19-25 years | 25,302 | 4,661 (18.4) | 7,475 (29.5) | 7,769 (30.7) | 697 (2.8) | 3,397 (13.4) | 1,004 (4.0) | 299 (1.2) |
| 26-35 years | 84,661 | 20,715 (24.5) | 24,605 (29.1) | 29,437 (34.8) | 2,307 (2.7) | 5,622 (6.6) | 1,563 (1.8) | 412 (0.5) |
| 36-45 years | 135,140 | 41,337 (30.6) | 39,153 (29.0) | 40,599 (30.0) | 4,055 (3.0) | 7,215 (5.3) | 2,246 (1.7) | 535 (0.4) |
| 46-55 years | 145,732 | 53,231 (36.5) | 40,800 (28.0) | 37,321 (25.6) | 5,120 (3.5) | 6,832 (4.7) | 2,010 (1.4) | 418 (0.3) |
| 56-65 years | 112,059 | 51,874 (46.3) | 27,938 (24.9) | 23,088 (20.6) | 4,340 (3.9) | 3,719 (3.3) | 932 (0.8) | 168 (0.1) |
| 66-75 years | 47,738 | 28,155 (59.0) | 9,579 (20.1) | 7,197 (15.1) | 1,698 (3.6) | 869 (1.8) | 215 (0.5) | 25 (0.1) |
| ≥76 years | 17,884 | 13,849 (77.4) | 2,044 (11.4) | 1,323 (7.4) | 454 (2.5) | 173 (1.0) | 39 (0.2) | 2 (0.0) |
| **Sex** |  |  |  |  |  |  |  |  |
| Men | 173,624 | 65,934 (38.0) | 42,326 (24.4) | 44,024 (25.4) | 4,144 (2.4) | 12,868 (7.4) | 3,553 (2.0) | 775 (0.4) |
| Women | 402,187 | 149,037 (37.1) | 111,317 (27.7) | 104,449 (26.0) | 14,754 (3.7) | 16,541 (4.1) | 4,852 (1.2) | 1,237 (0.3) |
| Total | 575,811 | 214,971 (37.3) | 153,643 (26.7) | 148,473 (25.8) | 18,898 (3.3) | 29,409 (5.1) | 8,405 (1.5) | 2,012 (0.3) |

*Note.* The table shows the observed distribution of selected primary presenting motives in the analytical sample used for the main multinomial logistic regression model. Percentages are row percentages. The analytical sample included contacts with complete data on selected primary presenting motive, sex, age group, and calendar year. These values are descriptive and unadjusted; adjusted associations are reported in the main multinomial regression model, and adjusted predicted probabilities are reported separately.
