# Supplementary material for: Loneliness and emotional support helpline use in Spain: a 20-year observational study
Source: Front Psychol. 2026 Jul 13;17:1852702. doi: 10.3389/fpsyg.2026.1852702 (PMC13402487; doi:10.3389/fpsyg.2026.1852702)
Supplement: Supplementary file 3 [file Table_3.doc]

**Supplementary Table S3**

*Adjusted Predicted Probabilities of Selected Primary Presenting Motive by Sex and Age Group*

| Sex | Age group | N | Loneliness / communication difficulties | Depressed mood | Anxiety-related problems | Grief / bereavement | Suicidal ideation | Suicidal crisis | Suicide act in progress |
| --- | --- | --- | --- | --- | --- | --- | --- | --- | --- |
| Men | ≤18 years | 2,442 | 15.94 | 24.28 | 21.94 | 2.22 | 26.80 | 6.54 | 2.27 |
| Men | 19-25 years | 9,938 | 18.94 | 26.48 | 29.12 | 2.09 | 17.12 | 4.93 | 1.33 |
| Men | 26-35 years | 30,712 | 25.67 | 26.65 | 33.45 | 2.07 | 9.14 | 2.44 | 0.58 |
| Men | 36-45 years | 43,257 | 32.21 | 26.37 | 28.92 | 2.26 | 7.50 | 2.25 | 0.48 |
| Men | 46-55 years | 42,001 | 38.76 | 25.28 | 24.51 | 2.61 | 6.63 | 1.86 | 0.34 |
| Men | 56-65 years | 28,182 | 49.60 | 22.22 | 19.50 | 2.74 | 4.65 | 1.11 | 0.17 |
| Men | 66-75 years | 11,864 | 62.44 | 17.73 | 14.10 | 2.56 | 2.52 | 0.60 | 0.06 |
| Men | ≥76 years | 5,228 | 79.99 | 10.00 | 6.69 | 1.81 | 1.24 | 0.27 | 0.01 |
| Women | ≤18 years | 4,853 | 15.66 | 30.00 | 24.79 | 3.56 | 19.11 | 4.87 | 2.01 |
| Women | 19-25 years | 15,364 | 18.09 | 31.52 | 31.73 | 3.19 | 11.04 | 3.35 | 1.09 |
| Women | 26-35 years | 53,949 | 23.78 | 30.44 | 35.52 | 3.10 | 5.22 | 1.51 | 0.43 |
| Women | 36-45 years | 91,883 | 29.82 | 30.20 | 30.57 | 3.35 | 4.32 | 1.38 | 0.36 |
| Women | 46-55 years | 103,731 | 35.62 | 29.10 | 26.05 | 3.88 | 3.90 | 1.18 | 0.26 |
| Women | 56-65 years | 83,877 | 45.18 | 25.84 | 20.97 | 4.25 | 2.87 | 0.74 | 0.14 |
| Women | 66-75 years | 35,874 | 57.83 | 20.84 | 15.40 | 3.89 | 1.59 | 0.40 | 0.05 |
| Women | ≥76 years | 12,656 | 76.39 | 12.02 | 7.69 | 2.84 | 0.86 | 0.20 | 0.01 |

*Note.* Values are model-based adjusted predicted probabilities expressed as percentages. N = 575,811. Probabilities were obtained from the main seven-category multinomial logistic regression model including sex, age group, and calendar year as predictors. Values represent average model-predicted probabilities within each sex-by-age stratum. The outcome categories are mutually exclusive and sum approximately to 100% within each row. Calendar year was included in the model but is not shown in the table.
