# Supplementary material for: Loneliness and emotional support helpline use in Spain: a 20-year observational study
Source: Front Psychol. 2026 Jul 13;17:1852702. doi: 10.3389/fpsyg.2026.1852702 (PMC13402487; doi:10.3389/fpsyg.2026.1852702)
Supplement: Supplementary file 4 [file Table_4.doc]

**Supplementary Table S4**

*Sensitivity Analysis Testing the Sex-by-Age-Group Interaction*

| Model | Outcome categories | N | Interaction included | AIC | BIC | -2 Log likelihood | Nagelkerke R² |
| --- | --- | --- | --- | --- | --- | --- | --- |
| Six-category main-effects model | 6 | 573,799 | No | 16,887.318 | 18,463.723 | 16,607.318 | .113 |
| Six-category interaction model | 6 | 573,799 | Sex × age group | 15,940.573 | 17,911.079 | 15,590.573 | .114 |

**Likelihood-ratio test for interaction**

| Effect | χ² | df | p |
| --- | --- | --- | --- |
| Sex × age group | 1,016.745 | 35 | <.001 |

*Note.* The six-category sensitivity model excluded suicide act in progress. Although the sex-by-age-group interaction improved model fit statistically, the increase in Nagelkerke R² was minimal (.113 to .114). Therefore, the seven-category main-effects model was retained as the primary model, and the interaction analysis was treated as a sensitivity analysis.
